# Antibody-mediated neutralization of ACBP/DBI has anorexigenic and lipolytic effects

**DOI:** 10.1080/21623945.2020.1736734

**Published:** 2020-03-11

**Authors:** Valentina Sica, Isabelle Martins, Omar Motiño, José M. Bravo-San Pedro, Guido Kroemer

**Affiliations:** a Metabolomics and Cell Biology Platforms, Gustave Roussy Cancer Campus, Villejuif, France; b INSERM U1138, Centre de Recherche des Cordeliers, Sorbonne Université, Université de Paris, Paris, France; c Team “Metabolism, Cancer & Immunity”, équipe 11 Labellisée Par la Ligue Contre le Cancer, Paris, France; d Pôle de Biologie, Hôpital Européen Georges Pompidou, AP-HP, Paris, France; e Suzhou Institute for Systems Medicine, Chinese Academy of Sciences, Suzhou, China; f Karolinska Institute, Department of Women’s and Children’s Health, Karolinska University Hospital, Stockholm, Sweden

**Keywords:** Adiposity, anorexia, appetite, autophagy, obesity

## Abstract

We recently identified acyl coenzyme A-binding protein (ACBP)/diazepam binding inhibitor (DBI) as a novel ‘hunger factor’: a protein that is upregulated in human or murine obesity and that, if administered to mice, causes hyperphagy, adipogenesis and obesity. Conversely, neutralization of ACBP/DBI by systemic injection of neutralizing monoclonal antibodies or autoantibodies produced after auto-immunization against ACBP/DBI has anorexigenic and lipolytic effects. Thus, neutralization of ACBP/DBI results in reduced food intake subsequent to the activation of anorexigenic neurons and the inactivation of orexigenic neurons in the hypothalamus. Moreover, ACBP/DBI neutralization results into enhanced triglyceride lipolysis in white fat, a surge in free fatty acids in the plasma, enhanced incorporation of glycerol-derived carbon atoms into glucose, as well as an increase in β-oxidation, resulting in a net reduction of fat mass. Importantly, ACBP/DBI neutralization also stimulated an increase in autophagy in various organs, suggesting that it might mediate anti-ageing effects.

Obesity is the most prevalent pathological condition worldwide. Although epidemiological factors such as exaggerated carbohydrate intake and sedentary life style could constitute a basis for obesity prevention, the actual prevalence of obesity (40% of the adult US population) [1] calls for novel therapeutic interventions. Indeed, obesity has become the most important risk factor for the accelerated manifestation of age-related pathologies including cardiovascular disease and cancer [2].

Why would obesity accelerate the ageing process? Caloric excess leads to suppression of autophagy [2–6], which is the most important cytoplasmic rejuvenation mechanism. Upon endogenous or exogenous stress, portions of the cytoplasm including damaged organelles, misfolded protein aggregates or intracellular pathogens can be sequestered in autophagosomes for their subsequent degradation and recycling in lysosomes. It is well established that induction of autophagy by caloric restriction (CR) or so-called CR mimetics (CRMs) has a positive impact on health span and lifespan [3,5,6], suggesting that the obesity-related suppression of autophagy might, on the contrary, have negative effects on organismal health and longevity.

Based on the general rule that cellular stress is communicated from within the cell to the extracellular microenvironment or the entire organism [7], we recently started the search for factors that are secreted from cells in an autophagy-dependent fashion. We found that various human and mouse cell types release acyl coenzyme A-binding protein (ACBP)/diazepam binding inhibitor (DBI) upon induction of autophagy into the extracellular space. Moreover, a 24-hour fasting period caused an elevation of circulating ACBP/DBI levels in the plasma of mice [8–10].

We subsequently discovered that intraperitoneal or intravenous injection of recombinant ACBP/DBI protein into mice was sufficient to induce an immediate (within less than 30 min) hyperphagic response with activation of orexigenic neurons and the inhibition of anorexigenic centres in the hypothalamus [8]. These effects are likely to be indirect, mediated through peripheral metabolic (rather than central nervous) effects of ACBP/DBI. Indeed, ACBP/DBI injection causes the rapid upregulation of glucose transporters in hepatocytes, stimulates glucose uptake into the liver and white adipose tissue, and entails a partial reduction (by approximately 25%) of circulating glucose levels. In conditions of a glucose clamp, the hyperphagic response induced by ACBP/DBI injection is lost, and the activation of orexigenic neurons is suppressed [8]. This latter finding suggest that the partial reduction in glucose levels induced by ACBP/DBI is required for the orexigenic effects of this protein, arguing in favour of a peripheral (rather than direct central-nervous) action of ACBP/DBI.

Of note genetic evidence obtained in yeasts, nematodes and mice supports the notion that ACBP/DBI stimulates food-seeking behaviour across the phylogenic treat, meaning that the ablation of the genes coding for ACBP/DBI inhibits sporulation (in *Saccharomyces cerevisiae*), pharyngeal pumping (in *Caenorhabditis elegans*) and food intake (in mice) [11]. Moreover, human obesity was found to be coupled to an increase in ACBP/DBI mRNA expression in white adipose tissue, as well as to an elevation in plasma ACBP/DBI levels [8]. These findings suggest that increased ACBP/DBI might be involved in the pathogenesis of obesity.

Intrigued by these findings, we designed two strategies to neutralize ACBP/DBI in mice. The first strategy consisted in the generation of a monoclonal antibody (mAb) capable of recognizing and neutralizing ACBP/DBI in an acute fashion [8]. The second strategy involved repeated immunizations of mice with a recombinant ACBP/DBI protein coupled to the carrier keyhole limpet hemocyanine (KLH) together with a strong adjuvant, thus breaking self-tolerance to ACBP/DBI and inducing the production of autoantibodies. This procedure allowed for the long-term neutralization of ACBP/DBI [8]. Both the supplementation of exogenous antibodies and the induction of anti-ACBP/DBI autoantibodies yielded similar metabolic effects on mice (Figure 1).

**Figure 1.**
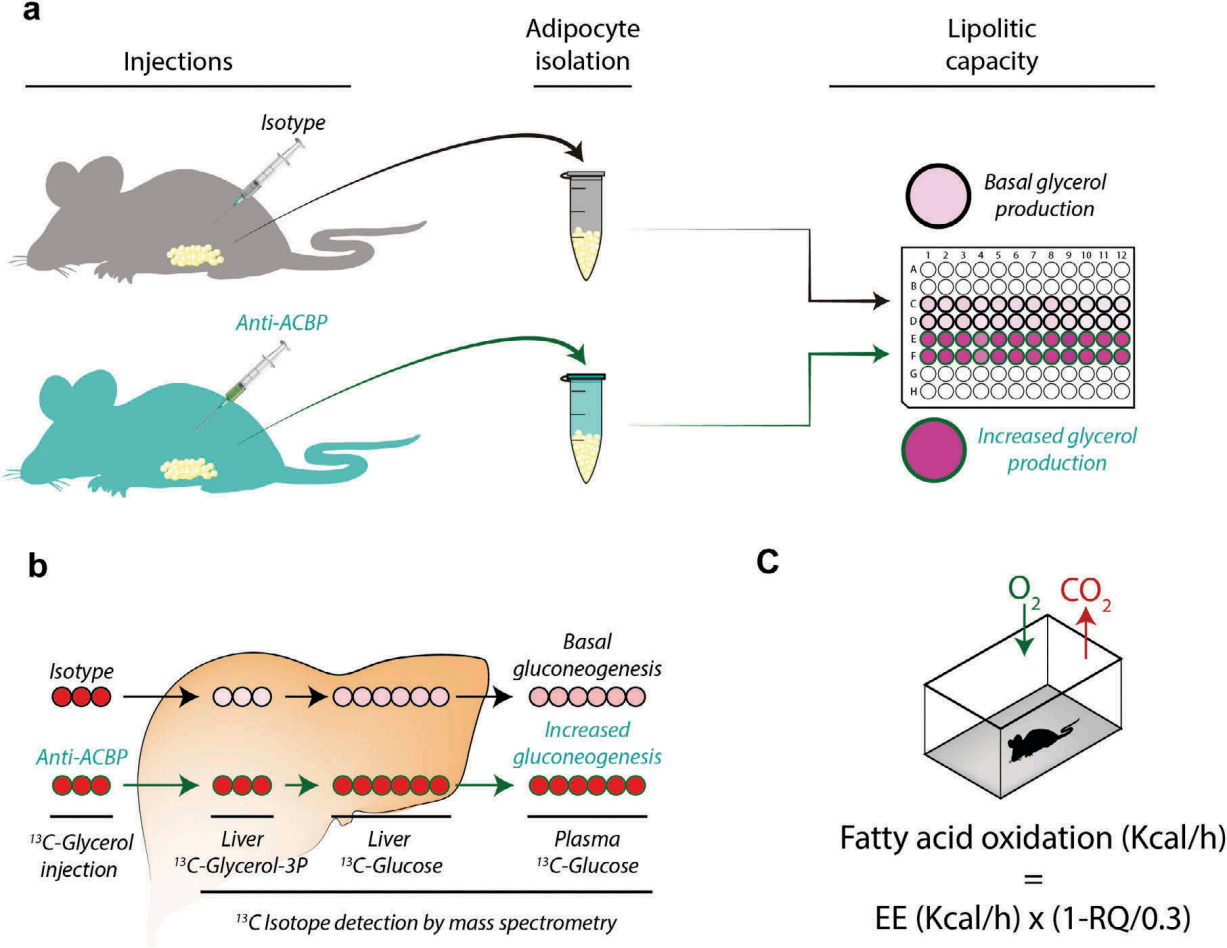
Induction of lipolysis *in vivo* after injection of neutralizing ACBP/DBI antibodies. Four to six hours after intraperitoneal injection of anti-ACBP antibody or a control antibody, the lipolytic activity was measured as glycerol production by white adipose tissues (a), by injection of ^13^ C-glycerol followed by mass spectrometric quantification of ^13^ C-glycerol-3-phosphate in hepatocytes and ^13^ C-glucose in liver and plasma (b), or whole body respirometry (c). Energy expenditure (EE) and oxygen consumption and carbon dioxide production (RQ = vCO_2_/vO_2_) were used to calculate fatty acid oxidation

ACBP/DBI neutralization strongly reduced the hyperphagic response induced by transient starvation (24 h). As shortly as 30 min after intraperitoneal injection of an anti-ACBP/DBI mAb, the activation of orexigenic neurons was inhibited, suggesting that these effects are mediated by the neutralization of peripheral (not central-nervous) ACBP/DBI because an antibody is expected should cross the brain blood barrier [8]. In fed mice, ACBP/DBI neutralization caused a transient and mild increase in glucose levels (by about 20%) coupled to the inhibition of glucose uptake by the liver and white adipose tissue [8,12]. Moreover, ACBP/DBI inhibition resulted in a dramatic effect on lipid metabolism. The white adipose tissue from mice receiving neutralizing ACBP/DBI antibodies exhibited an increase in lipolysis, as measured ex vivo 4–6 hours post-injection (Figure 1(a)). This was coupled to an enhanced conversion of glycerol into glucose, as determined by fluxomic measurements in which ^13^C-labelled glycerol was injected into mice and the abundance of ^13^C-containing glycerol metabolites and glucose was quantified in the liver (Figure 1(b)). ACBP/DBI injection also caused an increase in the plasma levels of free fatty acids. Moreover, whole body respirometry led to the conclusion that fatty acid oxidation was increased in conditions of ACBP/DBI neutralization (Figure 1(c)). Of note, ACBP/DBI neutralization also stimulated autophagic flux in a variety of organs including liver and white adipose tissue [8,9].

Altogether these data support the contention that ACBP/DBI neutralization results in lipo-catabolic reactions, commensurate with the observation that ACBP/DBI neutralization resulted in a reduction of fat mass in multiple different models, including age-associated weight gain of mice kept on a normal diet, high-fat diet-induced obesity, as well as genetically determined obesity of leptin-deficient Ob/Ob mice [8]. ACBP/DBI neutralization did not affect the lean mass of the mice. In contrast, long-term neutralization by ACBP/DBI by autoantibodies resulted in browning of white adipose tissue [8].

Altogether, these findings support the idea that ACBP/DBI might constitute an interesting target for treating obesity. It is our hope that clinical-grade ACBP/DBI mAbs will be developed and tested for the treatment of obesity and its comorbidities.
